# Examining the impact of urban compactness on work and social life disruption during COVID-19 pandemic: evidence from Jakarta, Indonesia

**DOI:** 10.1007/s12076-023-00347-7

**Published:** 2023-05-30

**Authors:** Usep Nugraha, Budy P. Resosudarmo, Rus’an Nasrudin

**Affiliations:** 1 Department of Economics, Faculty of Economics and Business, Universitas Indonesia, Jakarta, Indonesia; 2 Arndt-Corden Department of Economics, Crawford School of Public Policy, Australian National University, Canberra, ACT 2601 Australia; 3 Statistics Indonesia, Jakarta, Indonesia

**Keywords:** COVID-19, Urban form, Work and social life, Developing countries, I31, R12, R20

## Abstract

The COVID-19 pandemic has hit urban areas particularly hard, yet there is a lack of research on the hypothesis that living in more compact cities can provide better support for work and social conditions during the pandemic. This study addresses this gap by examining whether city compactness can mitigate the negative impact of the pandemic on the work and social life of urban residents in Jakarta, Indonesia. The study uses a household phone survey combined with publicly available urban form data. Ordinary Least Squares (OLS) regression, supplemented with a matching technique to address potential selection bias, is employed. The results suggest that living in more compact locations can reduce the disruption to work and social life associated with COVID-19 in urban communities. This positive effect is particularly experienced by males, non-migrants, and individuals from wealthy families.

## Introduction

The COVID-19 pandemic has forced people to change their work habits and social interactions (Kumar et al. 2021; Olivia et al. 2020; Salin et al. 2020; Sheth 2020). This shift has occurred for two reasons. First, individuals are concerned about contracting the virus (Wang et al. 2021). Second, governments have implemented policies to restrict activities and reduce physical interactions in an effort to slow the spread of the virus (Gloster et al. 2020). These restrictions have made it difficult for people to travel freely. These two factors have compelled individuals to alter their patterns and habits of working and socializing. Sudden changes in routine can disrupt one's sense of comfort and familiarity, and at a more severe level, this disruption can be an early indicator of mental health issues (Rajoo et al. 2021).

Transitions in work patterns and social interactions are more noticeable in urban areas than in rural areas. This is because the spread of the COVID-19 virus is more prevalent in urban areas, leading to greater concern about infection among urban communities than among rural ones. Additionally, restriction policies tend to be more pronounced in urban areas because they have a higher concentration of businesses and entertainment zones, which are the primary targets for restrictions on economic and social activities.

This paper examines the relationship between urban compactness and disruptions in work and social life during the COVID-19 pandemic. In general, urban development patterns are classified into two categories: compact and sprawl. Compact environments are characterized primarily by high density and diverse land uses, whereas sprawl is characterized by low density and more specific land uses. Debates on which design is better have been ongoing for a long time (Evans 2003; Neuman 2005), often associated with the issue of sustainability. However, the discussion on how city compactness impacts work and social life during the pandemic is still limited.

This study aims to investigate whether living in more compact urban locations results in less disruption to work and social life during the COVID-19 pandemic. Specifically, we estimate the premium of residing in compact locations during the pandemic with regard to work and social life interruptions. Our research was conducted in Jakarta, Indonesia, a developing megacity with a population of 10 million in 2020. Jakarta had the highest number of COVID-19 cases in Indonesia since the pandemic began, accounting for around 20% of the country's cases as of 1 August 2020. The administrative cities within Jakarta exhibit various characteristics, with some functioning as government areas, some as business areas, and others as areas with many settlements, implying different degrees of compactness across Jakarta's subdistricts. We utilize the variation in city compactness to evaluate the effect of urban form on people's work and social life during the pandemic.

We constructed an urban form index using indicators of residential density, land use, and street accessibility as the measure of compactness. In addition, individual perceptions of work and social life disruption during the COVID-19 pandemic were used as the outcome variable in ordinary least squares (OLS) regressions, with the urban form composite index as the variable of interest. We recognize the potential endogeneity of urban form and social life or work. For example, if social interaction increases with compactness, a particularly sociable person may intentionally choose to live in a more compact area to find a community that aligns with their outgoing personality (Brueckner and Largey 2008). To address this sorting issue's endogeneity, we conducted propensity score matching in the pre-estimation step.

This paper makes three contributions to the existing literature. First, it provides additional evidence on the protective factors against the harmful impacts of the COVID-19 pandemic, which is an essential addition to current research on COVID-19 that mainly focuses on the impact caused by the pandemic on various socioeconomic indicators (Miao et al. 2021; Proto and Quintana-Domeque 2021; Salin et al. 2020).

Second, we highlight the importance of urban compactness as a spatial factor that can moderate the negative impact of the pandemic on social life, which is a novel contribution as compactness variables (such as density) are often used as explanatory variables for outcomes of virus spread (Carozzi et al. 2020; Hamidi et al. 2020). Only a limited number of studies have positioned urban compactness as a moderating factor for the socioeconomic impact caused by the pandemic.

Third, this study advances the empirical strategy from the existing literature by addressing endogeneity issues, such as selectivity or sorting issues, which have been a challenge in most studies on the links between urban form and socioeconomic indicators. We tackle this problem by employing a pre-estimation matching technique to create counterfactuals of people living in a compact city among the control group individuals in our dataset (Garrido-Cumbrera et al. 2018).

This study develops an urban compactness index using publicly available geographic information system data of Jakarta and integrates it with respondents' locational information from our phone survey conducted from 1 May to 30 June 2020. The survey elicits the work and social disruptions experienced due to COVID-19 among 342 randomly selected individuals in Jakarta. Our findings suggest that compact urban form can reduce the degree of work and social disruptions experienced by urban communities during the COVID-19 pandemic, particularly for males, non-migrants, and individuals from wealthy families. The remainder of this paper is organized as follows: review on people livelihood during the pandemic, data and variables, identification strategy, results, and conclusion.

## People’s livelihood, social life, and city compactness

The COVID-19 pandemic has resulted in a significant transformation in the way people work and socialize. With the fear of contracting COVID-19 and the government's restrictions on economic and social activities, many people have opted to work remotely using online tools, resulting in a significant reduction in travel and movements. Moreover, people have been hesitant to spend money, as they are uncertain about how long these restrictions will last, resulting in a reduction of consumption and slower economic activity. This, in turn, has led to a reduction in workers' income (Suryahadi et al. 2021).

The accommodation services industry, which heavily relies on travel and tourism, is among the most affected workers in urban areas, as people have diverted their tourism expenditures to other needs, directly impacting travel and tourism behaviour (Aiello et al. 2021; Batool et al. 2021; Gerwe 2021; Bratic et al. 2021). However, workers in other economic sectors have also felt the effects of the COVID-19 pandemic and the accompanying government policies, especially those with low incomes who are unable to do their jobs using electronic devices and the internet (Paul et al. 2021). Despite the aid programs provided by the government and non-government organizations to minimize the economic shock due to the pandemic, the negative impact on people's livelihoods has been disastrous.

Besides affecting livelihoods, the COVID-19 pandemic has also impacted the social relations of individuals and communities. Various societal activities that are commonly carried out within the community, such as local celebrations (e.g., religious holidays and wedding receptions) that are characteristic of Indonesian society, have been either eliminated or significantly reduced. This is because people are either concerned about the spread of the virus or are following lockdown measures, which has resulted in them keeping their distance from others.

The disruption of livelihoods and reduced intensity of neighbour interactions can potentially lead to mental health problems. Therefore, it is essential to identify potential mediators to better understand the underlying mechanisms. Despite the same harmful shock exposure, the intrinsic factors possessed by the individual can differentiate the stringency of the disruption felt by the individual. Factors such as individual characteristics (e.g., age and type of work), family characteristics, and environmental characteristics may play a role in this differentiation.

This study investigates how environmental factors, particularly the compactness of urban living, can impact livelihood and social life during the COVID-19 pandemic. Research suggests that compact living environments may lead to less social life disruption and better mental health outcomes due to the social capital they foster (Cabrera and Najarian 2015; Glaeser and Sacerdote 2000; Hemani et al. 2017). Compact living arrangements allow for more frequent interactions between individuals, whether in open public facilities or through chance encounters on the street. These interactions can strengthen the bonds between neighbors and foster a high level of social capital within a compact setting (Evans 2003; Neuman 2005).

However, it is important to note that overcrowded areas have the opposite effect. Areas that are too dense can make people uncomfortable, leading to a lower quality of social welfare. This feature is also noteworthy because during the COVID-19 pandemic, the spread of the virus occurred more quickly in densely populated areas. Additionally, compact urban settings that often cause congestion or pollution, which rarely occur in non-compact areas, can also disrupt work and social life. Therefore, the aim of this paper is to investigate the complex relationship between environmental living compactness, particularly urban compactness, and the extent of work and life disruption during the COVID-19 pandemic, taking into account both the positive and negative effects.

## Data and variables

For our case study, we focus on Jakarta, the capital city of Indonesia with a population of over 10 million people (BPS—Statistics Indonesia 2017). Jakarta is comprised of five administrative cities, with 42 subdistricts (*kecamatan*) classified as urban, and one administrative regency classified as rural. This study analyses the compactness of the 42 urban subdistrict areas in Jakarta. Moreover, Jakarta was the site of the first reported case of COVID-19 in Indonesia and became the epicenter of the virus spread within the country.

To construct the urban form index for our study, we use three indicators: residential density, land use, and street accessibility. These indicators are commonly referred to as the “three D’s” of urban form, which include density, diversity, and design (Cervero and Kockelman 1997; Ewing and Hamidi 2014). We obtain the data for these indicators from publicly available spatial data provided by OpenStreetMap Indonesia (OpenStreetMap.or.id). The spatial data is extracted from two time periods: before the COVID-19 pandemic and the most recently available data from 2017.

Residential density is calculated by dividing the number of residential buildings by the residential area size, as shown in Eq. (1). Higher residential density values indicate more compact areas.

1 $${residential\_density}_{i}=\frac{{number\_residential\_building}_{i}}{{residential\_areas}_{i}}$$

where *i* is an index for urban subdistricts.

The entropy index of area type measures land use diversity in Jakarta's subdistricts, as presented in Eq. (2). We differentiate between residential and non-residential areas. The entropy index value ranges from zero to one, with a value close to one indicating a balanced land use between residential and non-residential areas, which implies a more compact subdistrict. Conversely, a value closer to zero indicates a less compact subdistrict.

2 $${land\_use\_diversity}_{i}=\frac{-\left[{\sum }_{j=1}^{k}{P}_{ji}ln\left({P}_{ji}\right)\right]}{ln\left(k\right)}$$

where *j* is an index for area type, *k* is the number of area types (or, for our case, equals to 2), and ${P}_{ji}$ is the percentage of land use type *j* in area *i*. The higher the index means the more diverse land use the area is.^1^

Street accessibility is the third scale and is determined by the ratio of road length to area size, as shown in Eq. (3). Higher values of street accessibility indicate greater compactness.

3 $${street\_accessiblity}_{i}=\frac{{road\_length}_{i}}{{area}_{i}}$$

We present the statistics for the mean and standard deviation of the three urban form indicators in the 42 subdistricts of Jakarta in Table 1. The mean residential density in our sample is 35 units per hectare, which is considered medium–high density (greater than 30 units per hectare) according to Edwards and Turrent (2000) and Susanti et al. (2016). This density level can bring benefits such as energy efficiency, increased public transportation utilization, and mixed land use opportunities. However, it may also have drawbacks, such as a tendency toward higher unit costs and issues with crime and vandalism. Moreover, the high standard deviation indicates a wide variation in density between the subdistricts in Jakarta.

**Table 1:** Descriptive statistics of urban form indicators

Variable	Description	Mean	SD
Residential density	Number of residential buildings per residential area (unit per hectare)	35.303	13.131
Land use diversity	Share of residential land use in sub-district	0.699	0.192
Street accessibility	Road length (km per sq. km)	24.755	5.460

The average road length in our sample is 24.8 km per square km, which is slightly higher than that of Vancouver, the densest city in Canada, with 23.2 km per square km (Ye et al. 2018). Additionally, it is much higher than that of Bangkok, the capital city of Thailand, with 17 km per square km (Kamarajugedda and Lo 2019). The mean proportion of residential land use in Jakarta's subdistricts is 69.9%, which is lower than that of Mairena del Aljafre, a city in Sevilla, Spain, with residential land use comprising 74.4% of the city (Garrido-Cumbrera et al. 2018).

To calculate the urban form index, we employ the aggregation method by adding the three indicators. Residential density and street accessibility are standardized to ensure comparability, as shown in Eqs. (4) and (5):

4 $$std\_{residential\_density}_{i}=\frac{{residential\_density}_{i}-MIN\left(residential\_density\right)}{MAX\left(residential\_density\right)-MIN\left(residential\_density\right)}$$

where *MIN(residential_density)* is the lowest residential density value, and *MAX(residential_density)* is the highest residential density value in the subdistricts in the dataset.

5 $$std\_{street\_accessiblity}_{i}=\frac{{street\_accessiblity}_{i}-MIN\left(street\_accessiblity\right)}{MAX\left(street\_accessiblity\right)-MIN\left(street\_accessiblity\right)}$$

where *MIN(street_accessibility)* is the lowest street accessibility value, and *MAX(street_accessibility)* is the highest street accessibility value in the subdistricts in the dataset.

**Fig. 1:**
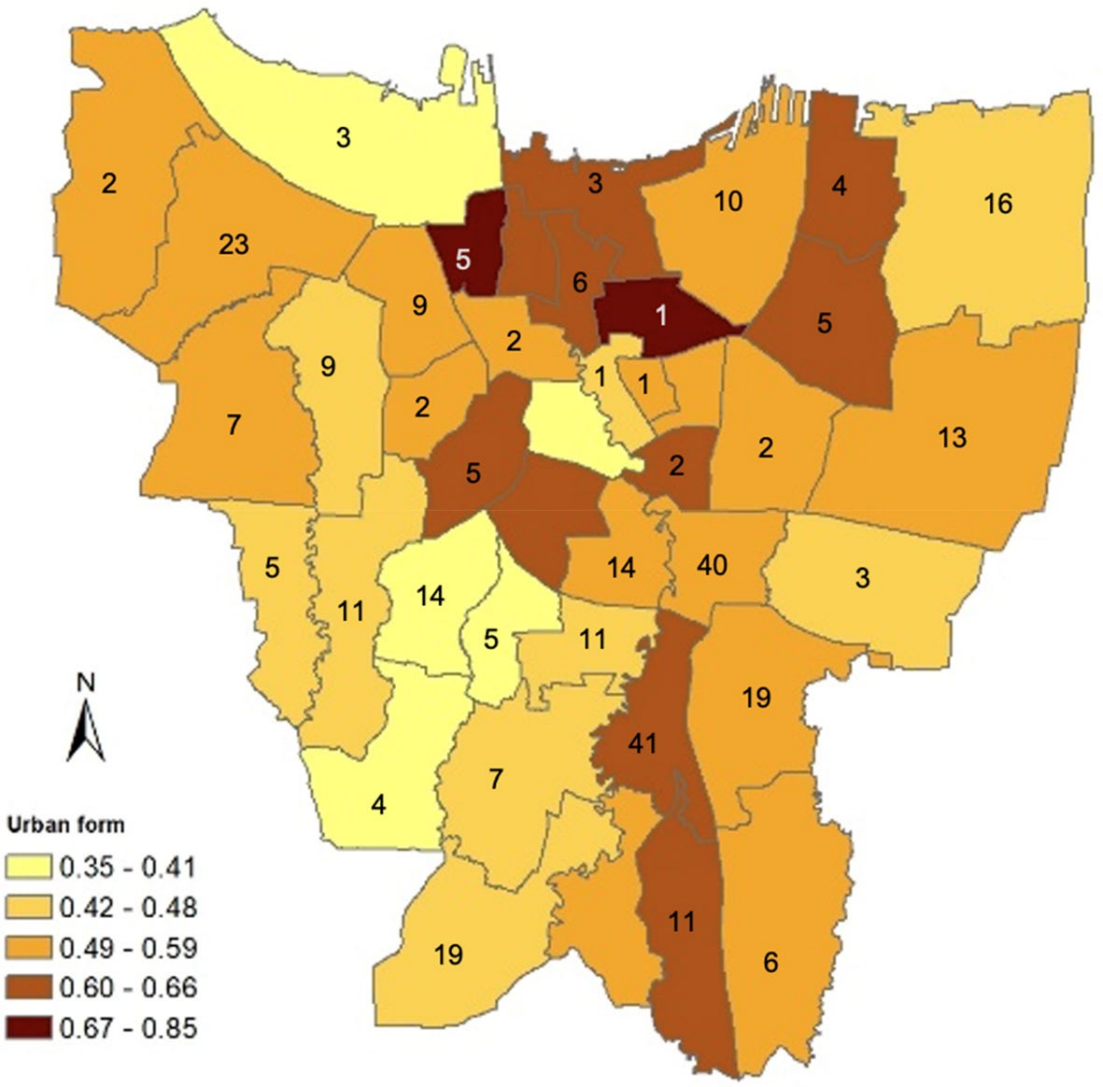
Distribution of urban form index and number of observations by subdistricts in Jakarta, 2020

The urban form index is the average of the standardized residential density, standardized street accessibility, and land use diversity indicators, assuming equal contribution from each of the three indicators. This index ranges from zero to one, where a higher value indicates a more compact area. The distribution of the urban form index is shown in Fig. 1, where darker areas represent more compact subdistricts. The map indicates a tendency for the compact subdistricts to be randomly placed, rather than concentrated only in the center of the city.

The second source of data was collected through a phone survey conducted between 1 May and 30 June 2020, in collaboration between the Faculty of Economics and Business at the University of Indonesia (FEB-UI) and the Australian National University (ANU). Undergraduate students primarily from Universitas Indonesia (UI) and Universitas Negeri Jakarta (UNJ) were employed as enumerators for the survey. The study was approved by the ethics committee of the Australian National University.

The sampling frame and sample weights for the phone survey were collected from the statistical agency of Indonesia (BPS), using the 2018 Flood Survey sampling frame and the Micro and Small Enterprise sampling frame of the 2016 Economic Census. A random sample of 4,600 respondents was selected to be interviewed by phone, but 3,559 could not be reached, and 699 declined to participate. Ultimately, only 342 respondents agreed to be interviewed, resulting in a response rate of approximately 7%. This response rate is comparable to other phone surveys conducted in Indonesia during the COVID-19 pandemic (Bella et al. 2022; The Ministry of Health et al., 2020). Among the 342 collected responses, 340 were processed, while the other two were excluded due to incomplete answers. The distribution of these 340 observations is shown in Fig. 1. Verbal recordings were obtained with respondents' consent before the survey questions were elicited, using computer-assisted software Survey Solution provided by the World Bank.

The phone survey focused on work and social life indicators, including working conditions and social relations during the COVID-19 pandemic. The survey also asked about social distancing measures, social assistance, job type, and family characteristics. To ensure the quality of responses, we limited the number of questions and kept the interview length to approximately 15 min.

The work and social life variable is measured by utilising straightforward questions, “How disrupted was your work, family life, and relationships with other people because of cases of virus transmission and deaths due to COVID-19?”. Respondents could select one of four answer options, “not disrupted”, “slightly disrupted”, “moderately disrupted”, or “severely disrupted”. The variable is coded to be zero to three. Higher scores indicate that individuals feel more disrupted.

In addition to individual-level variables, this study also examined community-level variables, specifically health access, which was proxied by the number of health facilities per square km area and obtained from the Village Potential Statistics (PODES) of Indonesia 2018. Table 2 presents the descriptive statistics for our data, including the average score of 1.872 for work and social life disruption in Jakarta during the early period of the pandemic. This score suggests that people felt moderately disrupted. Social distancing policies were well-enforced, with 74.8% of Jakarta's residents being aware of them and 80.3% implementing social distancing measures by reducing outdoor activities. The government's response to the pandemic was also seemingly successful, with 76% of respondents reporting that they had received government social assistance.

**Table 2:** Descriptive statistics of respondent characteristics (weighted)

Variables	Mean	SD	Min	Max
Work and social disruption	1.872	1.165	0	3
Urban form	0.548	0.086	0.35	0.85
Individual characteristics				
Age	44.348	15.215	18	72
Male = 1	0.579	0.494	0	1
Immigrant = 1	0.480	0.500	0	1
Job characteristics				
Working in the trade and accommodation sector = 1	0.408	0.492	0	1
Work hours higher than 40 h per week = 1	0.616	0.486	0	1
Family characteristics				
Family size	5.550	2.431	1	20
High expenditure family = 1	0.310	0.462	0	1
Social distancing implementation				
Knowing Social Distancing = 1	0.748	0.434	0	1
Doing Social Distancing = 1	0.803	0.398	0	1
Social assistance				
Social assistance from the government = 1	0.760	0.427	0	1
Social assistance from non-government = 1	0.236	0.425	0	1
Health access				
Hospital access (unit)	0.504	0.324	0.10	2.84
Community health centre access (unit)	0.664	0.435	0.20	2.11
Other health facilities access (unit)	7.707	4.935	2.02	19.02

## Estimation strategy

This study addresses the potential for systematic selection bias in the dataset before estimating the impact of urban form on work and social life disruptions, specifically the bias caused by sorting mechanisms. Sorting occurs when individual characteristics drive them to live in more compact or less compact subdistricts, which can influence the likelihood of individuals experiencing work and social life issues.

For example, if older individuals tend to live in less compact areas and have lower work-social life resilience due to their age, the estimates of the urban form effect on work-social life disruptions could be understated. If this issue is not appropriately addressed, it can lead to biased estimates (Duranton and Turner 2018).

We utilized a multiple linear regression of the urban form on pre-determined covariates listed in Table 2 to detect the possibility of systematic sorting. If significant associations between covariates and the urban form variable are found as the dependent variable, it suggests a sorting issue. To address this problem, we took two steps: first, we excluded outliers, and second, we used a matching method to overcome the issue.

To identify any potential outliers in the dataset, we used the interquartile range method. However, it is important to note that excluding outliers is not a precise method to control for sorting problems. For this method, we excluded observations with absolute values above the median by the interquartile range (Q3-Q1). The variables significantly associated with the urban form were subjected to the outlier removal process.

To address the sorting issue, we employed propensity score-based matching using the PSMATCH2 command in STATA software developed by Leuven and Sianesi (2003). The aim was to achieve a balanced condition where the characteristics of observations in compact areas are similar to those in non-compact areas. Propensity score matching creates an artificial control group by matching each observation in compact areas with an individual in non-compact areas who has similar observable characteristics. The matching approach is based on the counterfactual framework of Rosenbaum and Rubin (1983), which identifies non-treated units that are similar to the treated ones in the sample. We used various cutoff definitions of urban form measures to define treatment and control groups to ensure robustness.

The selection into urban form measure is specified as follows:

6 $$P\left(DU{F}_{i}=1|{{\varvec{Z}}}_{{\varvec{i}}}\right)=F(\widehat{{\varvec{\theta}}}{{\varvec{Z}}}_{{\varvec{i}}})$$

Here, $DU{F}_{i}$ is a dummy variable that takes the value of one for compact urban forms and zero for non-compact urban forms, with the cutoff value of 0.5 based on the urban form index. The alternatives and their resulting impacts are presented in the robustness section.

The vector ${Z}_{i}$ represents individual and community-level characteristics that serve as proxies for sorting issues, with $\widehat{\theta }$ as their coefficients. We posit that individuals with a higher propensity for experiencing work-social relation problems are more likely to choose compact locations based on their characteristics and the availability of healthcare on the supply side. The estimated value of $\widehat{\theta }$ in propensity score matching (PSM) is calculated using a probit model for limited dependent variables.

The primary specification for examining the impact of urban form on work and social life disruptions is presented in Eq. (7), where *i* is an index for observation and *j* is an index for subdistricts:

7 $${y}_{ij}=\alpha +\beta U{F}_{j}+\gamma {X}_{ij}+\delta {S}_{j}+{\varepsilon }_{ij}$$

The outcome variable ${y}_{ij}$ represents work and social life conditions, ${X}_{ij}$ defines individual-level covariates, and ${S}_{j}$ defines community-level covariates. $U{F}_{j}$ denotes the urban form index, which is the variable of interest. ${\varepsilon }_{ij}$ is the error term and is assumed to be white noise. $\beta$ is the parameter of interest used to measure the unit effect of urban form on work and social life. A negative and statistically significant value of *β* indicates that urban form is a protective factor for work and social life conditions during the COVID-19 pandemic.^2^ To estimate Eq. (7), this study employs ordinary least squares (OLS) with outlier removal and matching strategies.

The first post-regression analysis in this study aims to test the robustness of the treatment effect coefficient ($\beta$). The first robustness test examines whether the coefficient remains statistically significant within the bootstrapping confidence interval. We perform the bootstrapping confidence interval using the framework developed by Roodman et al. (2019).

The second robustness check is a coefficient stability test using Eq. 8 developed by Emily Oster (Oster 2019). The test evaluates the stability of the treatment effect coefficient ($\beta$) after controlling for observed and unobserved covariates. The bias-adjusted treatment effect (${\beta }^{*}$) is estimated using the coefficient with controls ($\widetilde{\beta }$), the coefficient without controls ($\dot{\beta }$), and the proportionality coefficient ($\delta$) that captures the relationship between the unobservable and observable controls and the treatment effect. We assume a maximum R-squared value (${R}_{max}$) that could be obtained by including all observed and unobserved covariates. Then, we calculate the R-squared values of the model with controls ($\widetilde{R}$) and without controls ($\dot{R}$). In this study, we report the value of $\delta$ from the Oster formula for various scenarios of the R-squared maximum, while assuming ${\beta }^{*}$ is equal to zero (Oster 2019).

8 $${\beta }^{*}\approx \widetilde{\beta }-\delta \left[\dot{\beta }-\widetilde{\beta }\right]\frac{{R}_{max}-\widetilde{R}}{\widetilde{R}-\dot{R}}$$

One consideration for implementing this test is to examine the impact of omitted variables on estimation bias. This is particularly relevant for unobservable factors that were not included in the main model but could have influenced the decision to live in a compact environment and affected work-social life status during the COVID-19 pandemic, potentially leading to bias. For example, individual income is an unobservable factor that may have caused bias in this study, as those with higher incomes may tend to live in certain areas and have better work-social living conditions during the pandemic due to their ability to save money or access treatment.

The third test for robustness involves assessing the treatment effect coefficient using data generated from multiple matching methods, alternative cutoff values for distinguishing compact and non-compact urban areas, and the entropy balance method (Hainmueller and Xu 2013; Tübbicke 2022) as an additional means of achieving balanced conditions.

The fourth robustness test is to test the sensitivity of our estimates towards possible measurement errors in work-social life conditions and urban form variables. First, we perform sensitivity analysis to assess the possibility of measurement errors in the work-social life indicator. The original measurement of work and social status is based on a specific question with four categorical answers, which is intended to make the interview process easier but raises doubts about whether the same answers indicate the same work-social status among observations. We transform these four categories of work and social life variables into binary (disrupted and undisrupted). The marginal effect of urban compactness in this sensitivity analysis is estimated using OLS and logit estimators to observe whether they indicate a similar effect to the results from the main estimate.

Secondly, we use the ordered logit model to evaluate the marginal effect of urban form on work and social life, which has an ordinal scale. Thirdly, we also examine the sensitivity of the urban form variable by transforming it into a binary variable indicating compact or not compact areas. This transformation is done because the main estimation uses post-matching results where the matching process is based on binary transformed urban form variables.

The final robustness test is related to the possibility of spatial dependencies because we study the effect of urban form from a geographical standpoint. Therefore, we also examine the stability of the urban form effect using the spatial error model and spatial lag model. To make the spatial model estimation work, we expand the data based on the weight of the matching results. The spatial weighting matrix is created based on rook and queen contiguity.

We conducted additional analyses to investigate which component of urban form is most relevant to work-social disruption. Specifically, we used Eq. (7) but replaced the urban form variable with its component variables, namely residential density, land use entropy, and road density. Additionally, we extended the analysis by examining the heterogeneity effect of urban form on work and social life during the COVID-19 pandemic, exploring potential differences based on variables such as gender, migrant status, and family expenditure.

## Results

### Pre-estimation

In this section, we assess the possibility of data sorting based on residing in a compact urban environment. The analysis indicates that four variables are significantly associated with the urban form variable, suggesting the presence of sorting issues. Two variables are at the individual level, including age and family size, and the other two variables are at the community level, including access to community health centres and other health facilities. The results in Table 3, column "Full data" show that young and large families tend to live in compact subdistricts. Moreover, good health access is more likely to be found in more compact subdistricts, as indicated by the two health facility variables that describe the ease of access to health.

**Table 3:** Identifying the association between urban form and covariates

Urban form	Full data		Excluded outlier		Matched data	
Individual characteristics						
Age	− 53.60***	(12.08)	− 34.23*	(18.23)	− 36.89	(22.79)
Male	− 0.96	(0.60)	− 0.95	(0.86)	− 1.42	(0.85)
Immigrant	− 1.03	(0.68)	− 1.15	(1.04)	− 1.39	(0.93)
Job characteristics						
Working in Trade and Accommodation Sector	− 0.59	(0.59)	− 0.11	(0.90)	− 0.65	(0.99)
Work hours higher than 40 h per week	0.79	(0.57)	0.88	(0.97)	0.36	(1.10)
Family characteristics						
Family size	6.43**	(2.26)	0.21	(2.56)	2.25	(3.20)
High Expenditure Family	− 0.42	(0.53)	0.01	(0.81)	0.85	(0.64)
Social distancing implementation						
Knowing social distancing	0.91	(0.69)	1.21	(1.09)	1.48	(1.00)
Doing social distancing	− 0.11	(0.95)	1.16	(0.85)	0.94	(0.67)
Social assistance						
Social assistance from government	0.64	(0.40)	0.76	(0.67)	0.39	(0.59)
Social assistance from non-government	0.41	(0.59)	0.72	(0.52)	0.87	(0.53)
Health access						
Hospital access	0.07	(0.67)	0.97	(0.80)	0.43	(0.78)
Community health centre access	2.59***	(0.88)	1.24	(0.93)	0.09	(0.57)
Other health facilities access	19.02**	(7.36)	9.48	(10.62)	3.90	(5.10)
Observations	340		314		271	

^***^, **, and * indicate 1, 5, and 10% of statistical significance. Standard errors clustered at the subdistrict level are in parentheses

We examined the presence of outliers in the observational data of the four variables significantly associated with the urban form variable. Outliers were found in the family size, community health centre access, and other health access variables, except for the age variable. A total of 26 outliers were identified, resulting in 314 observations remaining in the dataset. Next, we re-evaluated the association between urban form and these four variables using the excluded outlier data. The results showed that the associations of the three variables were no longer significant. However, the age variable still had a significant association with weaker significance (Table 3, columns “Excluded outlier”).

**Fig. 2:**
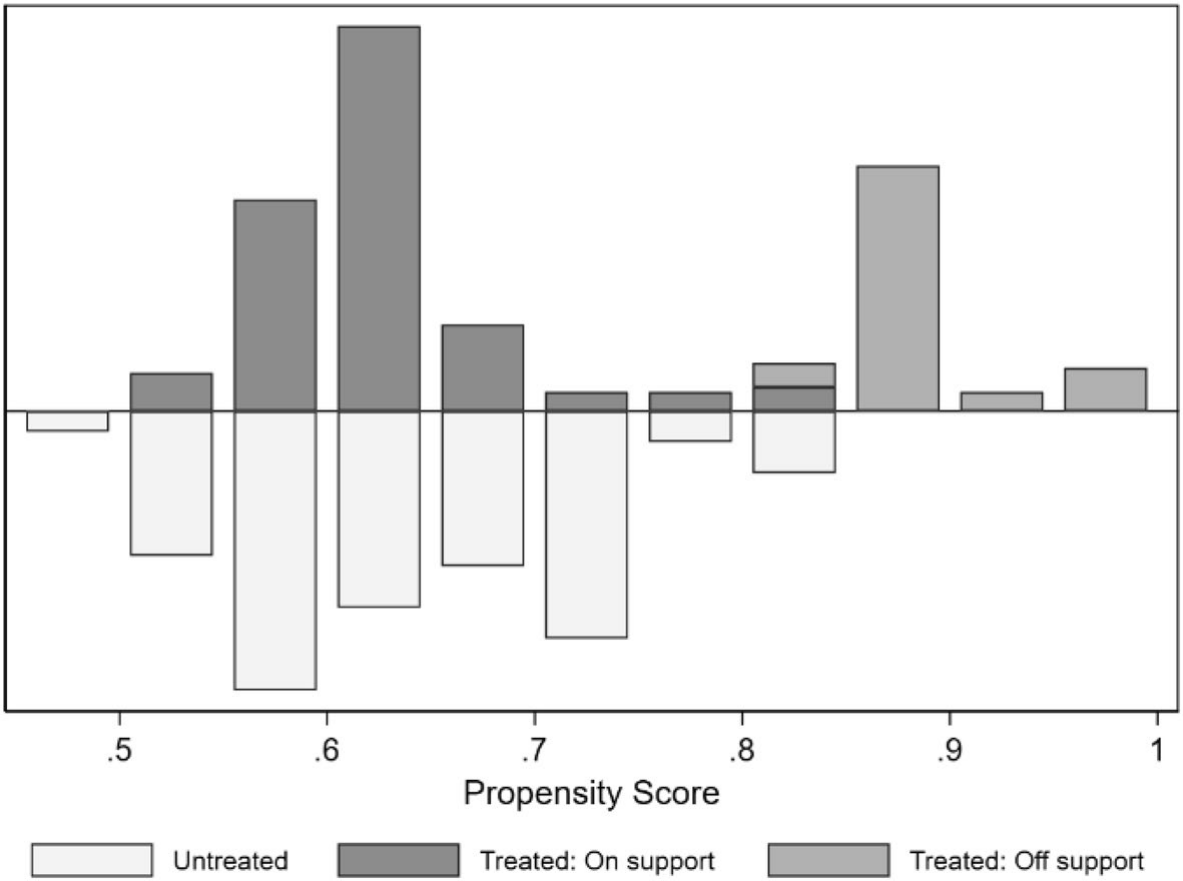
Propensity scores matching graph

Propensity score matching is used to obtain more balanced conditions, and the results of the probit regression and propensity graph can be found in Table 4 and Fig. 2, respectively. Observations without a counterfactual pair (off-support) are dropped, and after several iterations, the matching method of kernel with uniform distribution is chosen to obtain a balanced data set (Table 3, columns “Matched data”). The matching process resulted in the discarding of several observations, leaving a final sample of 271 observations. As a result of the matching, there are no longer any covariates significantly associated with the urban form variable.

**Table 4:** Probit estimate of the selection to urban compactness

Variables	Mean	Standard error
Age	− 0.00	(0.01)
Family size	0.01	(0.03)
Community health centre access	− 0.02	(0.30)
Other health facility access	0.10***	(0.03)

^***^, **, and * indicate 1, 5, and 10% of statistical significance respectively

### Urban form effect on work and social life during COVID-19

Table 5 reports the results of OLS regressions on Eq. (7) using the full and excluded outlier datasets in columns labeled "Full data" and "Excluded outlier," respectively. However, the main estimate of Eq. (7) is obtained by using matched data and incorporating weights obtained from the propensity scores matching, as shown in the column labeled "Matched data" in Table 5. The urban form coefficient in the matched data is − 2.80 and statistically significant at a 5% level, indicating that individuals who live in compact urban environments have less disruption to work and social life during the COVID-19 pandemic. The magnitude is more significant than in the complete data and excluded outlier data, which were − 2.38 and − 2.70, respectively, suggesting possible positive bias due to sorting. However, there is a decrease in R-squared in the excluded outlier data and matched data, which is likely due to the reduction in the number of observations resulting in less variation that covariates can explain.

**Table 5:** Relationships between urban form and the work and social life disruption

Work-social disruption	Full data	Excluded outlier	Matched data
Urban form	− 2.38**	− 2.70**	− 2.80**
	(1.04)	(1.19)	(1.12)
Individual characteristics	Yes	Yes	Yes
Job characteristics	Yes	Yes	Yes
Family characteristics	Yes	Yes	Yes
Social distancing implementation	Yes	Yes	Yes
Social assistance	Yes	Yes	Yes
Health access	Yes	Yes	Yes
Constant	Yes	Yes	Yes
Observations	340	314	271
R^2^	0.310	0.252	0.261

^***^, **, and * indicate 1, 5, and 10% of statistical significance respectively. Standard errors clustered at the subdistrict level are in parentheses

The economic significance of the effect can be explained by interpreting the magnitude as the marginal effect of a one standard deviation increase in the urban form variable, which is 0.086 (see Table 2). The corresponding change is a reduction of the work and social life disruption score by 0.24 in the matched data. This implies a reduction of 13% in the mean value of the outcome variable.

The direction and magnitude of the urban form effect in our study is not directly comparable to previous related studies as we are the first to use the urban form index (with Ewing’s approach) as a factor affecting work and social life during the COVID-19 outbreak. If the outcome variable of our study can also be viewed as a potential proxy for well-being, a possible comparison can be made with the work of Huerta and Utomo (2021) who analyse the effect of urban green spaces on changes in subjective well-being conditions during the pandemic in Mexico City. They found that people who often use urban green spaces have 1.7 points better subjective well-being than those who do not. This is in line with our results that suggest that people living in more compact areas face less work and social life disruptions (which can also be viewed as better subjective well-being) than those living in not compact areas. However, it is not clear whether the magnitude of the urban form effect in our study is higher than the urban green spaces effect in Huerta and Utomo’s study.

Outside the COVID-19 pandemic, other relevant studies that created an urban form index using Ewing’s approach include Garrido-Cumbrera et al. (2018) and Sturm and Cohen (2004), but both studies found insignificant effects of urban form on mental health.

### Robustness

The stability of the urban form's effect on work and social life disruption is examined through several robustness tests, including different standard error formulas, coefficient stability testing, different matching methods, and alternative operational definitions of the outcome and interest variables. The matched data are used in conducting these tests and subsequent analysis. Firstly, the bootstrapping confidence interval is performed using the framework developed by Roodman et al. (2019). The results show that the coefficient remains statistically significant within the bootstrapping confidence interval, indicating a robust urban form effect (Table 6). The absence of a zero value in the confidence interval range suggests that the estimation confirms the stability of hypothesis testing, even with a small sample size.

**Table 6:** Bootstrap confidence interval

Variables	t-stat	Prob	95% Confidence Interval	
			Lower bound	Upper bound
Urban form	− 2.496	0.04	− 5.432	− 0.1655

Note Bootstrap specification: wild, nonnull, mammen weight, 999 replications. All specification includes all relevant covariates

Second, another robustness test was conducted using the coefficient stability framework developed by Oster (2019) to address omitted variable issues. The maximum assumed R-squared values were set at 1.3, 1.5, and 1.7 times the given R-squared, and the results of the test can be seen in Table 7. When the R-squared maximum was set to 1.3 times, the framework produced a delta value of − 4.578, which made the beta value not significantly different from zero (This indicates that the urban form effect is not significant). The value suggests that unobservable variables would need to be weighted 4.5 times more important to make the observable effects on the urban form insignificant. Therefore, our estimate produces a robust regression coefficient, and it appears that the unobserved covariates are not that important.

**Table 7:** Coefficient stability testing

	Controlled (*R*^2^ max = 1.3 × *R*^2^)	Controlled (*R*^2^ max = 1.5 × *R*^2^)	Controlled (*R*^2^ max = 1.7 × *R*^2^)
Delta	− 4.578	− 3.178	− 2.433

Third, we conducted a robustness test using different matching methods (see Table 10 in the Appendix). We did not pay attention to whether covariates correlated with the urban form variable after matching. We used radius matching with callipers 0.025 and 0.01 as alternatives. These alternatives resulted in more observations being discarded than the matching method used in the pre-estimation. The other options were one-nearest neighbour, five-nearest neighbour, ten-nearest neighbour, and epanechnikov kernel with common support. All urban form coefficient values were negative and statistically significant, although they tended to produce larger magnitudes, the differences with our main coefficient were not substantive.

We used a cut-off value of 0.5 to conduct the matching process of kernel with uniform distribution on the urban form variable. The cut-off value determined whether a subdistrict was included in the treatment or control group. The cut-off was not a value from a particular rule or standard but was trivial by taking the middle value of 0 and 1. Therefore, we evaluated the robustness of the effect by shifting the cut-off to 0.45 and 0.55. The results showed that changing the cut-off did not significantly revise the urban form coefficient (Table 11 in the Appendix).

We also used the entropy balance method by Hainmueller and Tübbicke as an alternative to the matching method (Hainmueller and Xu 2013; Tübbicke 2022). The entropy balance method has the same objective as propensity score matching, which is to balance observations between treatment and control groups. The matching was done by selecting counterfactuals from each observation in the treatment groups on control groups with similar propensity scores (established on certain matching determinants). In comparison, entropy balance was done by eliminating correlations between a treatment variable and certain covariates. Furthermore, two entropy balancing methods were used. The first was the entropy balance method based on binary treatment variables raised by (Hainmueller and Xu 2013). The dummy urban form variable, as in the main matching process, was utilized again in this approach. The second was the entropy balance method established on continuous treatment variables introduced by (Tübbicke 2022) as the latest extension of Hainmueller and Xu’s methodology. All urban form coefficient values were negative and statistically significant in these alternatives. In addition, the coefficient values were not much different from our main matched data (Table 12 in the Appendix).

The fourth robustness test examines the sensitivity of our estimates towards possible important measurement errors in work-social life conditions and urban form variables. The first part of this test involves a sensitivity analysis of the effects of urban form on different scale specifications for the work and social life disruption variable. The original question to assess work-social disruption has four response options: “not disrupted”, “slightly disrupted”, “moderately disrupted”, or “severely disrupted”, which are coded as 0, 1, 2, and 3, respectively. We transformed these response categories into binary options, where 0 represents “not disrupted” and 1 represents “slightly, moderately, or severely disrupted”. Additionally, we also transformed the response categories into two other options, where 0 represents “not or slightly disrupted” and 1 represents “moderately or severely disrupted”. The results of using these different scales to measure work and social life disruption for estimating Eq. (7) using ordinary least squares (OLS) and maximum likelihood (logit) are presented in Table 13 in the Appendix. For the logit model, we report the average marginal effects. The coefficients obtained using the alternative scales of work and social life disruption have the same direction and comparable magnitude, and are statistically significant at a 5% level. Therefore, the consistency of the direction and magnitude of the effects using different measurements of the outcome variables supports the finding that living in compact areas is associated with lower work and social life disruption due to COVID-19.

The second part of this robustness test involved putting the work and social life disruption variable back to its original four ordinal scales, ranging from “not disrupted” to “severely disrupted”. However, we employed an ordered logit model to re-evaluate the effect of urban form on work-social disruption, and the results are presented in Table 14 in the Appendix. The model shows a significant positive marginal effect on the “not disrupted” category and a negative effect on the “severely disrupted” category, indicating that compactness increases the probability of not being severely disrupted and decreases the probability of being severely disrupted. An increase of one standard deviation of the urban form variable (0.086) increases the probability of not being disrupted by 5.7 percentage points in relative terms, while it decreases the probability of being severely disrupted by 9.5 percentage points. These results are consistent with the direction of effects found in the previous robustness tests.

The third sensitivity analysis involves modifying the urban form variable into a binary variable, where 1 represents compact and 0 represents not compact. The results, including the ordered logit model, can be found in Table 15 in the Appendix. The linear regression estimation shows a negative and significant effect, indicating that individuals are less disrupted in compact subdistricts than in non-compact ones. In addition, the ordered logit model's marginal effects on not disrupted and severely disrupted are significant and have the same direction as the OLS estimates. Once again, the results demonstrate that individuals residing in compact areas have a 10 percentage point higher likelihood of not being disrupted and a 17 percentage point lower likelihood of being severely disrupted due to the COVID-19 pandemic.

The last robustness check examines the possibility of spatial dependencies affecting the stability of our model. As the urban form variable analysed is at the regional level, we mitigate this issue by performing a spatial regression using a contiguity-based spatial weights matrix. We expand the matched data based on the weights produced from the propensity score matching procedure. However, expanding the data by weight on the data as is would result in millions of rows, so we limit the data expansion to yield a new data set of 926 rows (from the initial data set of 340). The comparison of the coefficients using the OLS model and spatial model can be seen in Table 16 in the Appendix. The spatial models used are the Spatial Error Model (SEM) and Spatial Lag Model (SLM). The results show that all urban form coefficients have significant negative values with magnitudes that are comparable to the main regression. Interestingly, the coefficients with spatial lag in the spatial models are significant, indicating the presence of spatial dependencies. However, we do not further discuss this issue in this paper. The spatial analysis aims to show the stability of the urban form effect on work and social life. Based on these results, we conclude that the urban form coefficient in our main regression is quite stable.

### Extended analysis

The first additional analysis aims to identify which component of urban compactness is relevant to work and social life disruption. The results are presented in Table 8. The regression estimates for the effect of each urban form component are generally consistent with our main findings. Prior to matching, only street accessibility has a significant negative coefficient. After using the matched data, both land use entropy and street accessibility show negative and significant coefficients, while residential density remains insignificant. Thus, the components of urban compactness that are most likely to be important in mitigating work and social disruption during the COVID-19 pandemic are land use diversity and street access.

**Table 8:** The effects by urban form components

Work-social disruption	Residential density		Land use entropy		Street accessibility	
	Pre-matching	Post-matching	Pre-matching	Post-matching	Pre-matching	Post-matching
Coefficient on work-social disruption	0.00 (0.01)	0.00 (0.01)	− 1.27 (0.95)	− 2.21** (0.92)	− 0.07* (0.04)	− 0.09** (0.04)
All covariates	Yes	Yes	Yes	Yes	Yes	Yes
Constant	Yes	Yes	Yes	Yes	Yes	Yes
*N*	340	271	340	271	340	271
R^2^	0.295	0.235	0.305	0.266	0.319	0.279

^***^, **, and * indicate 1, 5, and 10% of statistical significance respectively. Standard errors clustered at the subdistrict level are in parentheses. Data used in the post-matching column is equal to matched data in the main estimate

The second extended analysis examines the heterogeneous effects of urban compactness on work and social life disruption by performing regressions on subgroups based on gender, migrant status, and family expenditures. The results show that the effect of urban form is significant for males, non-migrants, and high family expenditure groups. However, the effect is insignificant for females, migrants, and low-family expenditure groups (see Table 9 in the Appendix). This suggests that the benefits of living in compact areas during the pandemic are not equally distributed across different subgroups of the population.

The heterogeneity analysis suggests that social capital may be an essential factor in explaining the different effects of urban form on work and social life disruption, particularly for the social life component. In Jakarta, community activities such as community meetings and neighbourhood security systems (siskamling) are often used as indicators of social capital. However, these activities tend to be dominated by males, which may explain why the effect of compact living environments is more strongly felt by males than females. Additionally, non-migrants may experience a stronger effect of urban form on work and social life disruption because their social cohesiveness is more affected by the environment in which they have lived. On the other hand, the urban form effect is less influential in low-expenditure families, as they may not have the same level of participation in social and community activities as high-expenditure families.

These assumptions are based on the theoretical relationship between compact urban form, social capital, and work and social life disruption outlined by Evans (2003). However, further research is needed to confirm this relationship in the specific context of Jakarta, as our study does not have relevant data to support these assumptions.

## Conclusion

This paper explores the role of urban form in moderating the negative effects of the COVID-19 pandemic on work and social life. Our findings suggest that urban form can serve as a protective factor in preventing these disruptions among individuals living in cities. Specifically, our estimates indicate a non-trivial premium of living in a more compact location, with a less reduction in work and social life disruptions by approximately 13%. Our conclusion is supported by several robustness tests that address potential selection bias, omitted variable bias, and spatial interdependency.

Our research supports the implementation of pro-compact city policies that prioritize balanced and diverse land use factors, as well as accessibility. Compactness can provide a protective layer for work and social life during times of crisis, such as the COVID-19 pandemic. Policymakers should focus on promoting the advantages of compactness by creating policies that prioritize these factors. By doing so, they can help build resilient communities and reduce the negative impacts of future crises.

Our research highlights the importance of land use diversity and road access in providing protection to communities against work and social life disruption during the pandemic. However, we do not deny the role of higher residential density in providing some protection in this matter as well. This research emphasizes the crucial role of good urban design in responding to urbanization and promoting the well-being of urban residents.

Our research also highlights the unequal distribution of benefits of compactness during pandemics. We found that certain subpopulations, such as males, non-migrants, and individuals from high-expenditure families, are more likely to benefit from living in compact environments during pandemics. This suggests the need for policymakers to consider targeted policy interventions that address these disparities. Specifically, we recommend that policymakers develop policies that prioritize the needs of females, migrants, and individuals from low-expenditure families. By doing so, they can help reduce the disparities in the benefits of compactness and build more inclusive communities that are better equipped to handle future crises. We believe that prioritizing equity and inclusion in urban policy can lead to stronger and more resilient communities that are better equipped to tackle future challenges.

In a broader context, our research emphasizes the positive impacts of compact city design on the economy, environmental quality, and social and health issues. Specifically, our findings suggest that compactness can help reduce work and social life disruptions, which may have potential benefits for mental health among individuals. This highlights the importance of prioritizing compact urban form in building stronger and more resilient communities that are better equipped to handle future crises.

While our research does not provide conclusive evidence on the mechanisms through which urban compactness protects against the negative effects of pandemics, we believe that stronger social capital may play a key role. Several studies have suggested that urban compactness is associated with stronger social capital (Glaeser and Sacerdote 2000; Cabrera and Najarian 2015; Hemani et al. 2017). Therefore, policymakers could consider an approach to urban policy that focuses on strengthening social ties within communities to mitigate the effects of future crises. However, the complex interplay between urban compactness, social capital, and community resilience is beyond the scope of this paper.

Overall, this paper provides important insights into the potential benefits of compact urban form in promoting resilience during crises. By knowing the role of urban form in mitigating the negative effects of pandemics, policymakers can develop more effective strategies for building strong and resilient communities. However, further research is needed to understand the mechanisms underlying these findings and to explore the potential trade-offs associated with promoting compact urban form.

## Appendix

See Tables

**Table 9:** The heterogeneous effects

Work-social disruption	Male group	Female group	Non-migrant	Migrant	Low family expenditure	High family expenditure
Urban form	− 5.20**	3.74	− 5.32***	− 0.02	− 0.25	− 6.05***
	(1.89)	(2.52)	(1.10)	(1.98)	(1.57)	(1.45)
All covariates	Yes	Yes	Yes	Yes	Yes	Yes
Constant	Yes	Yes	Yes	Yes	Yes	Yes
*N*	160	111	114	157	190	81
R^2^	0.443	0.342	0.428	0.263	0.187	0.665

^***^, **, and * indicate 1, 5, and 10% of statistical significance respectively. Standard errors clustered at the subdistrict level are in parentheses

9,

**Table 10:** The point estimates with different matching methods

	Radius (calliper = 0.025)	Radius (calliper = 0.01)	1-nearest neighbour (common support)	5-nearest neighbour (common support)	10-nearest neighbour (common support)	Epanechnikov kernel (common support)
Urban form	− 3.29***	− 3.55***	− 3.05***	− 3.33***	− 2.94**	− 2.92**
	(1.07)	(1.11)	(1.06)	(1.13)	(1.17)	(1.13)
*N*	265	258	214	245	252	271
R^2^	0.335	0.430	0.402	0.309	0.287	0.266

^***^, **, and * indicate 1, 5, and 10% of statistical significance respectively. Standard errors clustered at the subdistrict level are in parentheses. All specification includes all relevant covariates

10,

**Table 11:** The Matching point estimates with different cut-off

	Cut-off = 0.5 (main estimation)	Cut-off = 0.45	Cut-off = 0.55
Urban form	− 3.29***	− 3.43***	− 3.74***
	(1.07)	(1.21)	(1.13)
*N*	265	263	327
R^2^	0.335	0.291	0.345

^***^, **, and * indicate 1, 5, and 10% of statistical significance respectively. Standard errors clustered at the subdistrict level are in the parentheses. All specification includes all relevant covariates

11,

**Table 12:** Entropy balance estimates

	Entropy balance on binary treatment	Entropy balance on continuous treatment
Urban form	− 2.93***	− 2.14***
	(0.83)	(1.05)
*N*	340	340
R^2^	0.371	0.252

^***^, **, and * indicate 1, 5, and 10% of statistical significance respectively. Standard errors clustered at the subdistrict level are in parentheses. All specification includes all relevant covariates

12,

**Table 13:** Sensitivity analysis by different measures of work-social disruption

Work-social disruption (binary)	OLS		Logit model (Average marginal effects)	
	(Not disrupted = 0; others = 1)	(Not disrupted & slightly disrupted = 0; others = 1)	(Not disrupted = 0; others = 1)	(Not disrupted & slightly disrupted = 0; others = 1)
Urban form	− 1.25***	− 1.24**	− 1.08**	− 1.28**
	(0.33)	(0.53)	(0.43)	(0.60)
All covariates	Yes	Yes	Yes	Yes
Constant	Yes	Yes	Yes	Yes
Observations	271	271	271	271
R^2^	0.251	0.303		
Pseudo R^2^			0.276	0.278

^***^, **, and * indicate 1, 5, and 10% of statistical significance respectively. Standard errors clustered at the subdistrict level are in parentheses. All specification includes all relevant covariates

13,

**Table 14:** OLS and ordered logit estimates with continuous urban form index

Work-social life disruptions	OLS	Ordered logit model			
		Not disrupted	Slightly disrupted	Moderately disrupted	Severely disrupted
Urban form	− 2.80**	0.66*	0.37	0.09	− 1.10**
	(1.12)	(0.34)	(0.23)	(0.16)	(0.46)
All covariates	Yes	Yes	Yes	Yes	Yes
Constant	Yes	Yes	Yes	Yes	Yes
Observations	271	271			
R^2^	0.261				
Pseudo R^2^		0.126			

^***^, **, and * indicate 1, 5, and 10% of statistical significance respectively. Standard errors clustered at the subdistrict level are in parentheses

14,

**Table 15:** OLS and ordered logit model in binary urban form index (cut-off = 0.5)

Work-social disruption	OLS	Ordered logit model			
		Not disrupted	Slightly disrupted	Moderately disrupted	Severely disrupted
Urban form (binary)	− 0.42*	0.10*	0.06	0.01	− 0.17**
	(0.19)	(0.06)	(0.03)	(0.02)	(0.08)
All covariates	Yes	Yes	Yes	Yes	Yes
Constant	Yes	Yes	Yes	Yes	Yes
Observations	271	271			
R^2^	0.256				
Pseudo R^2^		0.124			

^***^, **, and * indicate 1, 5, and 10% of statistical significance respectively. Standard errors clustered at the subdistrict level are in the parentheses

15, and

**Table 16:** The stability check of urban form coefficients with spatial dependencies models

Work-social disruption	OLS	Spatial error model	Spatial lag model
Urban form	− 2.62***	− 3.32***	− 2.72***
	(0.46)	(0.47)	(0.47)
Spatial lag (error)		0.76*** (0.10)	
Spatial lag (dependent)			0.33*** (0.10)
Expanded observations	926	926	926
R^2^	0.202		
Pseudo R^2^		0.190	0.184

^***^, **, and * indicate 1, 5, and 10% of statistical significance respectively. Standard errors are in parentheses. Data is expanded proportionally based on the weight of the matching results in the main estimate. The expansion process is made because the spatial model used cannot adopt the available weights. The spatial weight matrix is based on contiguity. All covariates are used in the models

16.

## Footnotes

1 The index equals to 1 for a perfectly diverse area and closer to zero for a homogenous land use type area.

2 As the observations are divided into several subdistricts and the effect of urban form is measured at the subdistrict level, we address concerns about heteroscedasticity by applying the clustered standard error at the subdistrict level.

## References

[CR1] Aiello F, Bonanno G, Foglia F (2021). On the choice of accommodation type at the time of Covid-19. Some evidence from the Italian tourism sector. Curr. Issue Tourism.

[CR2] Batool M, Ghulam H, Hayat MA, Naeem MZ, Ejaz A, Imran ZE, Spulbar C, Birau R, Gorun TH (2021). How COVID-19 has shaken the sharing economy? An analysis using Google trends data. Econ. Res..

[CR3] Bella A, Swarnata A, Melinda G, Nurshadrina DS, Dartanto T (2022). Changes in smoking status and behaviors after the first 10 months of COVID-19 pandemic in Indonesia. Nicotine Tob. Res..

[CR4] BPS–Statistics Indonesia: Population Projection of Regency/City DKI Jakarta Province 2015–2025. Jakarta: BPS. (2017). https://jakarta.bps.go.id/publication/2017/12/27/1c9967923b29b829d906d436/proyeksi-penduduk-kabupaten-kota-provinsi-dki-jakarta-tahun-2015-2025.html

[CR5] Bratic M, Radivojevic A, Stojiljkovic N, Simovic O, Juvan E, Lesjak M, Podovšovnik E (2021). Should I stay or should I go? Tourists’ COVID-19 risk perception and vacation behavior shift. Sustainability.

[CR6] Brueckner JK, Largey AG (2008). Social interaction and urban sprawl. J. Urban Econ..

[CR7] Cabrera JF, Najarian JC (2015). How the built environment shapes spatial bridging ties and social capital. Environ. Behav..

[CR8] Carozzi, F., Provenzano, S., Roth, S.: Urban density and COVID-19. IZA Discussion Paper, No. 13440 (2020). https://docs.iza.org/dp13440.pdf

[CR9] Cervero R, Kockelman K (1997). Travel demand and the 3Ds: density, diversity, and design. Transp. Res. Part D: Transp. Environ..

[CR10] Duranton G, Turner MA (2018). Urban form and driving: evidence from US cities. J. Urban Econ..

[CR11] Edwards B, Turrent D (2000). Sustainable housing principles and practice. Taylor and Francis e-Library.

[CR12] Evans GW (2003). The built environment and mental health. J. Urban Health.

[CR13] Ewing, R., Hamidi, S.: Measuring sprawl. Smart Growth America (2014). https://smartgrowthamerica.org/wp-content/uploads/2016/08/measuring-sprawl-2014.pdf

[CR14] Garrido-Cumbrera M, Ruiz DG, Braçe O, Lara EL (2018). Exploring the association between urban sprawl and mental health. J. Transp. Health.

[CR15] Gerwe O (2021). The COVID-19 pandemic and the accommodation sharing sector: effects and prospects for recovery. Technol. Forecast. Soc. Chang..

[CR16] Glaeser EL, Sacerdote B (2000). The social consequences of housing. J. Hous. Econ..

[CR17] Gloster AT, Lamnisos D, Lubenko J, Presti G, Squatrito V, Constantinou M, Nicolaou C, Papacostas S, Aydın G, Chong YY, Chien WT, Cheng HY, Ruiz FJ, Garcia-Martin MB, Obando-Posada DP, Segura-Vargas MA, Vasiliou VS, McHugh L, Höfer S, Karekla M (2020). Impact of COVID-19 pandemic on mental health: an international study. PLoS ONE.

[CR18] Hainmueller J, Xu Y (2013). Ebalance: a stata package for entropy balancing. J. Stat. Softw..

[CR19] Hamidi S, Sabouri S, Ewing R (2020). Does density aggravate the COVID-19 pandemic. J. Am. Plann. Assoc..

[CR20] Hemani S, Das AK, Chowdhury A (2017). Influence of urban forms on social sustainability: a case of Guwahati. Assam. Urban Design Int..

[CR21] Huerta CM, Utomo A (2021). Evaluating the association between urban green spaces and subjective well-being in Mexico City during the COVID-19 pandemic. Health Place.

[CR22] Kamarajugedda, S.A., Lo, E.Y.M.: Modelling urban growth for Bangkok and assessing linkages with road density and socio-economic indicators. The international archives of the photogrammetry, remote sensing and spatial information sciences, XLII-4/W19, pp 255–262 (2019). https://doi.org/10.5194/isprs-archives-XLII-4-W19-255-2019

[CR23] Kumar P, Kumar N, Aggarwal P, Yeap JAL (2021). Working in lockdown: the relationship between COVID-19 induced work stressors, job performance, distress, and life satisfaction. Curr. Psychol..

[CR24] Leuven, E., Sianesi, B.: PSMATCH2: Stata module to perform full Mahalanobis and propensity score matching, common support graphing, and covariate imbalance testing (2003). https://ideas.repec.org/c/boc/bocode/s432001.html

[CR25] Miao J, Zeng D, Shi Z (2021). Can neighborhoods protect residents from mental distress during the COVID-19 pandemic? Evidence from Wuhan. Chinese Sociol. Rev..

[CR26] Mouratidis K (2017). Built environments and social well-being: How does urban form affect social life and personal relationship. Cities.

[CR27] Neuman M (2005). The compact city fallacy. J. Plan. Educ. Res..

[CR28] Olivia S, Gibson J, Nasrudin R (2020). Indonesia in the time of Covid-19. Bull. Indones. Econ. Stud..

[CR29] Oster E (2019). Unobservable selection and coefficient stability: theory and evidence. J. Bus. Econ. Stat..

[CR30] Paul A, Nath TK, Mahanta J, Sultana MN, Kayes ASMI, Noon SJ, Jabed A, Podder S, Paul F (2021). Psychological and livelihood impacts of COVID-19 on Bangladeshi. Asia Pacific J. Publik Heath.

[CR31] Proto E, Quintana-Domeque C (2021). COVID-19 and mental health deterioration by ethnicity and gender in the UK. PLoS ONE.

[CR32] Rajoo KS, Singh KD, Abdu A, Rosli Z, James GG (2021). Addressing psychosocial issues caused by the COVID-19 lockdown: Can urban greeneries help?. Urban Forest. Urban Green..

[CR33] Roodman D, MacKinnon JG, Nielsen MØ, Webb MD (2019). Fast and wild: bootstrap inference in stata using boottest. Stata J..

[CR34] Rosenbaum PR, Rubin DB (1983). The central role of the propensity score in observational studies for causal effects. Biometrika.

[CR35] Salin M, Kaittila A, Hakovirta M, Antillla M (2020). Family coping strategies during Finland’s Covid-19 Lockdown. Sustainability.

[CR36] Sheth J (2020). Impact of Covid-19 on consumer behavior: will the old habits return or die?. J. Bus. Res..

[CR37] Sturm R, Cohen DA (2004). Suburban sprawl and physical and mental health. Public Health.

[CR38] Suryahadi A, Al Izzati R, Yumna A (2021). The impact of Covid-19 and social protection programs on poverty in Indonesia. Bull. Indones. Econ. Stud..

[CR39] Susanti R, Soetomo S, Buchori I, Brotosunaryo PM (2016). Smart growth, smart city and density: in search of the appropriate indicator for residential density in Indonesia. Proc. Soc. Behav. Sci..

[CR40] The Ministry of Health, NITAG, UNICEF, WHO: COVID-19 Vaccine Acceptance Survey in Indonesia. (2020). https://www.unicef.org/indonesia/coronavirus/reports/covid-19-vaccine-acceptance-survey-indonesia

[CR41] Tübbicke S (2022). Entropy balancing for continuous treatments. J. Econ. Methods.

[CR42] Wang C, Tee M, Roy AE, Fardin MA, Srichokchatchawan W, Habib HA, Tran BX, Hussain S, Hoang MT, Le XT, Ma W, Pham HQ, Shirazi M, Taneepanichskul N, Tan Y, Tee C, Xu L, Xu Z, Vu GT, Kuruchittham V (2021). The impact of COVID-19 pandemic on physical and mental health of Asians: a study of seven middle-income countries in Asia. PLoS ONE.

[CR43] Ye C, Chen Y, Li J (2018). Investigating the influences of tree coverage and road density on property crime. Int. J. Geo-Inform..

